# Fas-Associated Factor 1 Promotes Hepatic Insulin Resistance via JNK Signaling Pathway

**DOI:** 10.1155/2021/3756925

**Published:** 2021-01-16

**Authors:** Bao Sun, Jiecan Zhou, Yongchao Gao, Fazhong He, Heng Xu, Xiaoping Chen, Wei Zhang, Ling Chen

**Affiliations:** ^1^Department of Clinical Pharmacology, Xiangya Hospital, Central South University, Changsha 410078, China; ^2^Department of Pharmacy, The Second Xiangya Hospital, Central South University, Changsha 410011, China; ^3^Institute of Clinical Pharmacy, Central South University, Changsha 410011, China; ^4^Institute of Clinical Medicine, The First Affiliated Hospital, University of South China, Hengyang 421001, China; ^5^Institute of Clinical Pharmacology, Central South University, Hunan Key Laboratory of Pharmacogenetics, Changsha 410078, China; ^6^Engineering Research Center of Applied Technology of Pharmacogenomics, Ministry of Education, Changsha 410078, China; ^7^National Clinical Research Center for Geriatric Disorders, Changsha 410008, China; ^8^Zhuhai People's Hospital (Zhuhai Hospital Affiliated with Jinan University), Zhuhai 519000, China; ^9^Department of Laboratory Medicine, National Key Laboratory of Biotherapy/Collaborative Innovation Center of Biotherapy and Cancer Center, West China Hospital, Sichuan University, Chengdu 610041, China; ^10^Department of Gastrointestinal Surgery, Xiangya Hospital, Central South University, Changsha, Hunan 410078, China

## Abstract

Fas-associated factor 1 (FAF1), a member of the Fas death-inducing signaling complex, is reported to interact potentially with diverse proteins and function in diverse cellular possesses. It remains unclear, however, whether FAF1 is involved in hepatic metabolic disorder and insulin resistance. This study aims to elucidate the role and the molecular mechanism of FAF1 in hepatic insulin resistance. Rats treated with high-fat diets are used as hepatic insulin resistance animal models. Quantitative real-time PCR, immunohistochemistry, and immunofluorescence assay are utilized to detect the FAF1 expression. The expression of relevant proteins is detected by Western blotting. We determine ROS production, lipid accumulation, and glucose uptake by using flow cytometry. Immunoprecipitation is employed to investigate protein-protein interaction. We find that increased expression of FAF1 occurred in the livers of insulin-resistant rats. Using gain-of-function and loss-of-function approaches, we observe dramatic exacerbation of insulin resistance, upregulated gluconeogenesis genes, downregulated glucose transport genes, and enhanced ROS production by FAF1 overexpression, whereas downregulation of FAF1 leads to a completely opposite phenotype. Mechanistically, FAF1 interacts directly with c-Jun N-terminal kinase (JNK) and activates its phosphorylation, thereby blocking the downstream insulin signaling pathway and leading to insulin resistance. Our data indicate that FAF1 is a potent regulator in hepatic metabolic disorder and insulin resistance.

## 1. Introduction

Insulin resistance, which is defined as impaired ability of cells, such as hepatocytes, adipocytes, and skeletal muscle cells to respond to the action of insulin, plays an essential role in the development of type 2 diabetes, obesity, and metabolic syndrome [1]. Dysregulation of glucose metabolism is a hallmark of insulin resistance in the hepatocytes, and the potential mechanisms mainly include decreased glycogen synthesis and failure to inhibit glucose production [2]. However, the elaborated mechanisms, including the complex molecular interactions and relevant cellular behaviors, involved in the process of metabolic disorder and hepatic insulin resistance are not yet fully understood.

Fas (CD95/Apo-1), a surface glycoprotein belonging to the tumor necrosis factor receptor family and a specific mediator of apoptosis [3], is constitutively expressed in most tissues. Accumulating evidence indicated that the Fas-meditated apoptosis pathway was a key bridge between obesity-associated fatty liver and increased susceptibility to liver damage [4–6]. Moreover, circulating soluble apoptotic marker Fas was positively with insulin resistance in the serum of newly diagnosed type 2 diabetes [7]. Myeloid cell-expressed Fas had an impact on inflammatory and glucose homeostasis, consequently contributing to the development of insulin resistance [8]. Recently, literature demonstrated that Fas regulated hepatic mitochondrial function and fatty acid oxidation, thus promoting the progression of hepatic steatosis and insulin resistance [9]. Although the relationship between Fas and insulin resistance has been widely studied, it remains unknown whether FAF1, a Fas-binding protein, may be involved in hepatic metabolic disorders and insulin resistance.

FAF1 is an evolutionarily conserved protein that contains multiple protein-interaction domains and is also identified as an interactor of Fas that enhances apoptosis [10]. Apart from apoptosis, FAF1 participated in the process of regulating necrosis via activation of poly(ADP-ribose) polymerase 1 (PARP1) [11]. Furthermore, FAF1 contributed to caspase activation, ROS generation, and JNK activation in dopaminergic neurons [12]. Previous studies usually considered that FAF1 acted as a tumor suppressor by arresting the cell cycle and suppressing the NF-*κ*B pathway [13–15]. Besides, FAF1 was also reported to regulate the chaperone activity of Hsp70 and the innate immunity signaling pathway [16–18] and might serve as a scaffolding protein that meditated protein degradation in the ubiquitin-proteasome pathway [19]. While it is well accepted that FAF1 has a role in various biological processes, its role in metabolic disorders and insulin resistance is unclear. Strikingly, recent studies found that *FAF1* rs17106184 was susceptibility loci associated with type 2 diabetes by genetic association analysis and validation [20, 21], but the specific mechanism was unclear. On this basis, it is worth further investigating whether FAF1 participates in the pathogenesis of metabolic disorders and insulin resistance-associated type 2 diabetes.

In the current study, we show that FAF1 overexpression dramatically exacerbates glucose and lipid metabolic disorder, as well as insulin resistance by contributing to JNK activation in hepatocytes. These novel findings highlight FAF1 as a key meditator of hepatic metabolic disorder and insulin resistance.

## 2. Materials and Methods

### 2.1. Animals and Treatments

Male rats were reared in a specific pathogen-free animal laboratory at the Experimental Animal Center of Central South University with a strictly controlled room temperature (22°C ± 2°C) and a 12 : 12-hour light-dark cycle. Six-week-old rats were adapted to the environment for 1 week and then fed a high-fat diet (HFD, 10% lard oil, 20% sugar, and 70% normal diet) or on a standard normal diet for 6 weeks with sufficient food and water. Afterwards, freshly prepared streptozotocin (STZ, Sigma-Aldrich, USA, 40 mg/kg) dissolved in a 0.1 M critic acid/sodium citrate buffer at pH 4.5 was intraperitoneally injected after 12 h of fasting in the rats with a high-fat diet. After two days, the rats' fasting blood glucose (FBG) was measured on several occasions, and the rats with an average FBG ≥ 11.1 mmol/L were considered as T2DM models. The animals were provided human care based on the Guide for the Care and Use of Laboratory Animals. The animal experiments were approved by the Animal Ethics Committee of Central South University (No. 2018sydw087).

### 2.2. Metabolic Analysis

Rats with a high-fat diet and those with a standard normal diet were injected with STZ and 0.1 M critic acid/sodium buffer, respectively. Two days later, all rats were fasted for 12 h per week, and their fasting blood glucose (FBG) was measured with a glucometer (ACCU CHEK Advantage, Germany). Serum insulin levels were analyzed with a rat insulin ELISA kit (Mercodia, Sweden). The homeostasis model of assessment-insulin resistance (HOMA-IR) was calculated using the following formula: FBG (mmol/L) × fasting serum insulin (FSI) (Ug/L)/22.5 [22]. Insulin resistance was considered in rats on a high-fat diet when the HOMA-IR of rats on a high-fat diet was significantly higher than that of rats on a normal diet. All protocols were performed according to the manufacturer's instructions.

### 2.3. Immunofluorescence and Immunohistochemical Analysis

Paraffin-embedded liver tissues were prepared for immunohistochemical analysis according to standard procedures [23]. Liver paraffin sections were dewaxed, and endogenous peroxidases were neutralized with 0.3% hydrogen peroxide. Liver sections were then blocked in 1% bovine serum albumin and incubated with the primary antibodies against FAF1 (ab183045, Abcam) overnight at 4°C, followed by incubation with secondary antibodies for 10 min. Subsequently, the sections were incubated for 30 min in 3,3′-diaminobenzidine tetrahydrochloride (Sigma-Aldrich) with hematoxylin counterstain. Images were visualized by the Leica DMI 4000B inverted microscope.

Immunofluorescence staining was performed as the method reported previously [24]. FAF1 (ab183045, Abcam, USA) antibody was diluted in 1% bovine serum albumin. The secondary antibody was fluorescein isothiocyanate-conjugated goat anti-rabbit antibody (Jackson ImmunoResearch, Westgrove, PA-EUA).

### 2.4. Quantitative Real-Time PCR

Total RNA was extracted from liver tissues or cultured cells with RNAiso Plus (Takara, China) and was reverse-transcribed into cDNA by a PrimeScript™ RT Reagent Kit (Takara, China) according to our previous study [25]. Primer sequences are shown in Supplementary Table 1.

### 2.5. Glycogen Content and Glucose Uptake Assay

Before the experiments, the cells were transferred and maintained in 6-well plates and then treated with FAF1 deletion or overexpression as well as the corresponding controls. After 2 hours of insulin stimulation, the glucose content was determined by the glycogen assay kit (BC0345, Solarbio, China). And the total protein amount of each well was measured using a BCA protein assay kit (Beyotime Biotechnology, China) to normalize the glucose consumption. Cellular glucose uptake was measured in cultured cells using a fluorescent 2-[N-(7-nitrobenz-2-oxa-1,3-diazol-4-yl)amino]-2-deoxy-d-glucose (2-NBDG) assay as previously described [26]. 10000 single-cell events were collected and analyzed by a flow cytometer (Beckman Coulter, USA).

### 2.6. Hepatic Lipid Accumulation Assay

Cultured cells were seeded into 6-well plates and then treated with FAF1 deletion or overexpression as well as the corresponding controls. Next, the cells were treated with palmitate (PA) or vehicle control for 24 hours. Then, the cells were incubated with 4,4-difluoro-1,3,5,7,8-pentamethyl-4-bora-3a,4a-diaza-s-indacene (BODIPY493/503) staining in the dark for 15 min at 37°C according to the protocol [27]. 10000 single-cell events were collected and analyzed by a flow cytometer.

### 2.7. ROS Determinations

ROS was measured in cultured cells using 2′,7′-dichlorofluorescin diacetate (10 *μ*M, DCFDA, Beyotime Biotechnology, China) and then was analyzed by a flow cytometer according to the manufacturer's instructions.

### 2.8. Cell Culture and Transfection

HepG2 cells and SK-HEP-1 cells were purchased from the Cell Bank of Chinese Academy, China Academy of Science. HepG2 cells were cultured in a humidified atmosphere (37°C, 5% CO_2_) in Dulbecco's modified Eagle's medium (DMEM) (Gibco, CA, USA) supplemented with 10% fetal bovine serum (FBS) (Gibco, CA, USA) and 1% penicillin/streptomycin (Thermo Fisher Scientific, Inc.). SK-HEP-1 cells were cultured in a humidified atmosphere (37°C, 5% CO_2_) in MEM supplemented with 10% FBS and 1% penicillin/streptomycin. To mimic insulin resistance model *in vitro*, PA (Sigma-Aldrich, USA) was added to the culture medium for 24 hours.

Two small interfering RNA (siRNA) against FAF1 and negative control were synthesized by RiboBio (Guangzhou, China) and listed in Supplementary Table 2. The plated cells were transfected with siRNA at a final concentration of 50 nM using Lipofectamine RNAiMAX reagent (Invitrogen, CA, USA) following the manufacturer's instruction. Human FAF1 cDNA was cloned into pcDNA3.1-Flag vector to generate the pcDNA3.1-Flag-FAF1 recombinant plasmids. Cells were transfected with 1.0 *μ*g plasmid per well of a 6-well plate using the ViaFect Transfection Reagent (Promega, USA) according to the manufacturer's instruction.

### 2.9. Western Blotting and Immunoprecipitation

HepG2 cells and SK-HEP-1 cells were lysed using RIPA lysis solution containing complete protease inhibitor and phenylmethylsulfonyl fluoride. The proteins were quantified using the BCA Protein Assay Kit (Beyotime Biotechnology, China). For immunoprecipitation, the cells were collected in RIPA buffer. The resulting lysates were precipitated with the relevant antibody and protein G-sepharose beads by incubation at 4°C overnight. Protein samples were separated by SDS-PAGE and transferred to PVDF membranes. The membranes were blocked with 5% skim milk and incubated with the relevant primary antibody. Immunostaining was detected with a Bio-Rad ChemiDoc XRS imaging system (Bio-Rad, USA).

The following antibodies were used. FAF1 (ab183045), JNK (ab179461), and GAPDH (ab181602) were obtained from Abcam. IRS1 (2382S), AKT (9272S), p-AKT (ser473, 4060S), GSK-3*β* (12456S), p-GSK-3*β* (5558S), and p-JNK (4668S) were purchased from Cell Signaling Technology. Flag (66008-3-Ig) and IgG (SA00001-1) were purchased from Proteintech.

### 2.10. Statistical Analyses

The data are represented as mean ± standard deviation, and at least 3 independent experiments were carried out. A two-tailed Student *t* test or one-way analysis of variance was used to determine significance. *P* values < 0.05 were considered statistically significant. SPSS version 22.0 (SPSS Inc., Chicago, IL) was used for analysis.

## 3. Results

### 3.1. Hepatic FAF1 Expression Is Increased in the Type 2 Diabetic Rat Model

**Figure 1:**
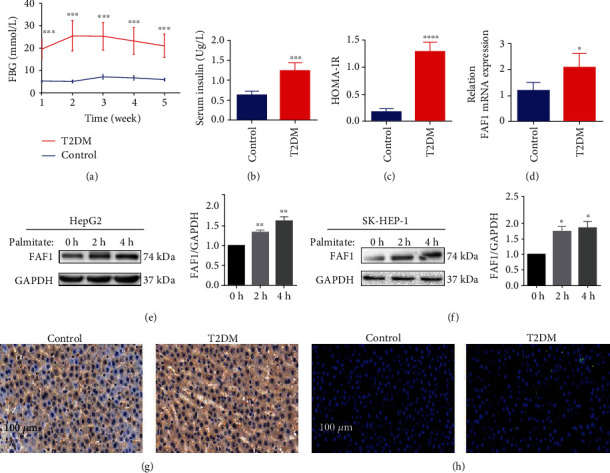
FAF1 expression was increased in type 2 diabetic rats of hepatic insulin resistance. (a) The fasting blood glucose (FBG) levels in the control and type 2 diabetes (T2DM) groups (*n* = 7 in each group). (b) The fasting serum insulin levels in control and T2DM, ^∗∗∗^*P* < 0.001 versus control. (c) The values of the homeostasis model of assessment-insulin resistance (HOMA-IR), ^∗∗∗∗^*P* < 0.0001 versus control. (d) The expression of FAF1 in the liver of type 2 diabetic rats, ^∗^*P* < 0.05 versus control. (e, f) The expression of FAF1 in PA-treated HepG2 and SK-HEP-1 cells, ^∗^*P* < 0.05 and ^∗∗^*P* < 0.01 versus control. (g, h) Representative immunohistochemistry and immunofluorescence staining of FAF1 in the liver tissue of type 2 diabetic rats.

As a first step towards elucidating the role of FAF1 protein in the regulation of glucose and lipid metabolism in the liver, we constructed the type 2 diabetic rat model that exhibited typical symptoms of insulin resistance, including the increased levels of FBG and FSI, as well as the values of HOMA-IR (Figures 1(a)–1(c)). Two days after the injection of STZ, FBG was greater than 11.1 mmol/L in all rats with a high-fat diet (Figure 1(a)). Moreover, HOMA-IR of all rats on a high-fat diet was significantly higher than that of rats on a normal diet, which suggested that insulin resistance occurred in all rats on a high-fat diet (Figure 1(c)). The expression levels of FAF1 mRNA were clearly increased in the livers of insulin-resistant rats (Figure 1(d)). Immunohistochemistry and immunofluorescence on liver sections further confirmed the significant elevation of FAF1 in the livers of insulin-resistant rats (Figures 1(g) and 1(h)). A previous study identified that PA (0.25 mM) induced insulin resistance without cell cytotoxicity in HepG2 cells [28]. To directly investigate whether FAF1 levels changed in hepatocytes exposed to a dynamic change under insulin resistance, HepG2 and SK-HEP-1 cells were exposed to PA (0.25 mM). The result showed that FAF1 expression was significantly increased in PA-treated cells (Figures 1(e) and 1(f)). In short, the expression of hepatic FAF1 protein was increased in the state of insulin resistance, thus suggesting a potential role of FAF1 in hepatic metabolism disorder.

### 3.2. FAF1 Expression Affects the Expression of Glucose Metabolism Genes

**Figure 2:**
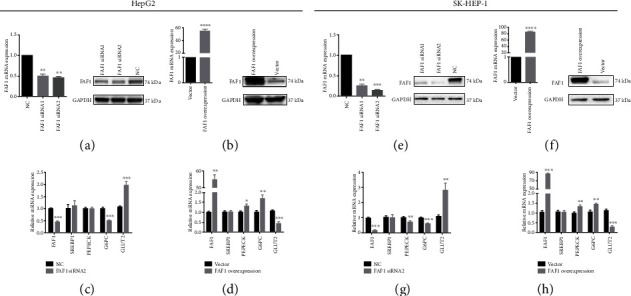
Overexpression and knockdown of FAF1 regulated the expression of glucose metabolism genes. (a, b) The mRNA and protein levels of FAF1 after knockdown and overexpression of FAF1 in HepG2 cells. (c) Knockdown of FAF1 downregulated *G6PC* expression but upregulated *GLUT2* expression in HepG2 cells. (d) Overexpression of FAF1 upregulated *PEPCK* and *G6PC* expression but downregulated *GLUT2* expression in HepG2 cells. (e, f) The mRNA and protein levels of FAF1 after knockdown and overexpression of FAF1 in SK-HEP-1 cells. (g) Knockdown of FAF1 downregulated *PEPCK* and *G6PC* expression but upregulated *GLUT2* expression in SK-HEP-1 cells. (h) Overexpression of FAF1 upregulated *PEPCK* and *G6PC* expression but downregulated *GLUT2* expression in SK-HEP-1 cells. All results are representative of three independent experiments. ^∗^*P* < 0.05, ^∗∗^*P* < 0.01, and ^∗∗∗^*P* < 0.001.

FAF1 was knockdown by two siRNAs (FAF1 siRNA1 and FAF1 siRNA2) and overexpressed by full-length human FAF1 plasmid. Since the FAF1 siRNA2 has better interference efficiency, we selected it for subsequent functional experiments. FAF1 expression was reduced about 60% and 80% of the negative control (NC) in HepG2 and SK-HEP-1 cells, respectively (Figures 2(a) and 2(e)), and its expression in FAF1-overexpressed HepG2 and SK-HEP-1 cells was increased to about 55 and 88 times compared with the vector, respectively (Figures 2(b) and 2(f)). Overexpression of FAF1 was able to activate the expression of gluconeogenic genes, including *PEPCK* and *G6PC*, but reduced the expression of *GLUT2* involved in glucose uptake in HepG2 and SK-HEP-1 cells (Figure 2(h)), whereas knockdown of FAF1 decreased the *PEPCK* and *G6PC* expression but increased *GLUT2* expression in SK-HEP-1 cells (Figure 2(g)). In HepG2 cells, FAF1 deletion only decreased the expression of *G6PC* but increased the expression of *GLUT2* (Figures 2(c) and 2(d)). Both FAF1 overexpression and deficiency did not affect the expression of *SREBP1* involved in lipogenesis. Collectively, FAF1 may mediate hepatic metabolism disorder by influencing the expression of glucose metabolism genes.

### 3.3. FAF1 Expression Affects Glycogen Synthesis, Glucose Uptake, and Lipid Accumulation

**Figure 3:**
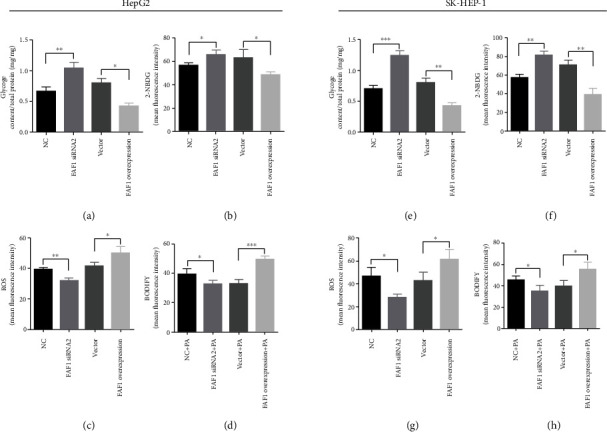
Overexpression and knockdown of FAF1 regulated glycogen synthesis, glucose uptake, ROS production, and lipid accumulation. (a, e) Knockdown of FAF1 increased insulin-stimulated glycogen synthesis, and overexpression of FAF1 decreased insulin-stimulated glycogen synthesis in HepG2 and SK-HEP-1 cells. (b, f) Knockdown of FAF1 promoted insulin-stimulated glucose uptake, and overexpression of FAF1 inhibited insulin-stimulated glucose uptake in HepG2 and SK-HEP-1 cells. (c, g) Knockdown of FAF1 reduced the ROS production, and overexpression of FAF1 increased the ROS production in HepG2 and SK-HEP-1 cells. (d, h) Knockdown of FAF1 ameliorated PA-induced lipid accumulation, and overexpression of FAF1 aggravated PA-induced lipid accumulation in HepG2 and SK-HEP-1 cells. Data represent means ± SD (*n* = 3 independent experiments). ^∗^*P* < 0.05, ^∗∗^*P* < 0.01, and ^∗∗∗^*P* < 0.001.

In line with the results of the influence of glucose metabolism genes, FAF1 overexpression inhibited insulin-stimulated glucose uptake and glycogen synthesis (Figures 3(a), 3(b), 3(e), and 3(f)). The fore result did not find that FAF1 affected the lipogenesis. Apart from lipogenesis, reduced lipid oxidation might also result in hepatic insulin resistance [29]. In order to determine whether FAF1 may impair mitochondrial fatty acid oxidation and increase lipid accumulation, we measured the influence of FAF1 overexpression on the PA-induced lipid accumulation. FAF1 overexpression aggravated PA-induced lipid accumulation (Figures 3(d) and 3(h)). In addition, ROS, mainly produced by mitochondria, have previously been proposed to be involved in insulin resistance [30]. Therefore, we also detected the levels of ROS and found that FAF1 overexpression increased ROS production (Figures 3(c) and 3(g)). Conversely, FAF1 deficiency enhanced insulin-stimulated glucose uptake and glycogen synthesis (Figures 3(a), 3(b), 3(e), and 3(f)). Furthermore, FAF1 deficiency decreased ROS production and ameliorated PA-induced lipid accumulation (Figures 3(c), 3(d), 3(g), and 3(h)). Taken together, these data demonstrate that FAF1 promotes glucose and lipid metabolic impairment.

### 3.4. FAF1 Overexpression Induces Insulin Resistance in Hepatocytes

**Figure 4:**
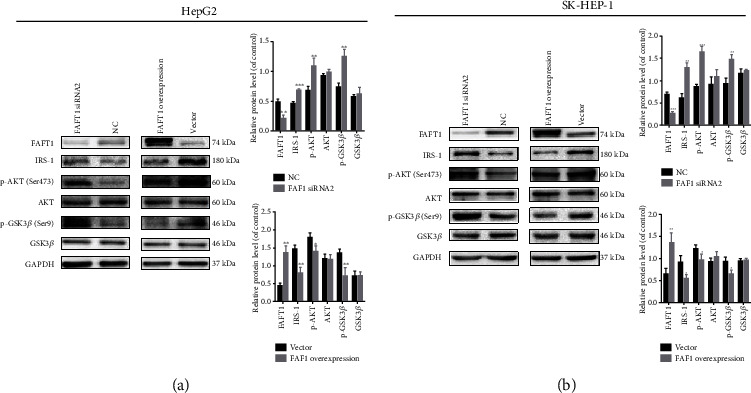
FAF1 overexpression induced insulin resistance in hepatocytes. (a) FAF1 deficiency activated the insulin signaling pathway, and FAF1 overexpression impaired the insulin signaling pathway in HepG2 cells. (b) Similar results were obtained in SK-HEP-1 cells. Data represent means ± SD (*n* = 3 independent experiments). ^∗^*P* < 0.05, ^∗∗^*P* < 0.01, and ^∗∗∗^*P* < 0.001.

Considering that FAF1 overexpression impaired glucose and lipid metabolic perturbation, we further explored the effect of FAF1 overexpression on hepatic insulin resistance. FAF1 overexpression decreased the expression of IRS1 and impaired the insulin signaling pathway in the HepG2 and SK-HEP-1 cells with insulin stimulation (Figures 4(a) and 4(b)). Inversely, FAF1 deletion increased the expression of IRS1 and promoted the activity of the insulin signaling pathway in the HepG2 and SK-HEP-1 cells with insulin stimulation (Figures 4(a) and 4(b)).

### 3.5. JNK Activation Is Required for the Effect of FAF1 on Insulin Resistance

**Figure 5:**
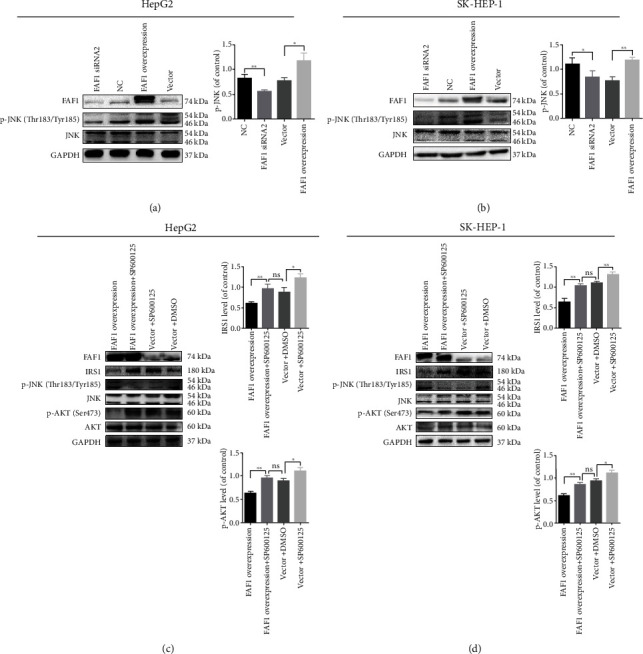
FAF1 regulated insulin signaling pathway via JNK. (a, b) FAF1 deficiency decreased JNK phosphorylation, and FAF1 overexpression increased JNK phosphorylation in HepG2 and SK-HEP-1 cells. (c, d) SP600125 (10 *μ*M), a JNK signaling inhibitor, restored the aberrant insulin signaling induced by FAF1 overexpression in HepG2 and SK-HEP-1 cells. Data represent means ± SD (*n* = 3 independent experiments). *ns*: not significant, ^∗^*P* < 0.05 and ^∗∗^*P* < 0.01.

JNK, one of the most investigated signal transducers in obesity and insulin resistance, was reported to promote insulin resistance via association with IRS1 [31, 32]. To elucidate the underlying mechanism that FAF1 overexpression decreased the expression of IRS1 and impaired the insulin signaling pathway, the JNK signaling pathway was studied. We observed that FAF1 overexpression increased JNK phosphorylation, whereas JNK phosphorylation was decreased in the FAF1 deficiency cell lines compared with their respective controls (Figures 5(a) and 5(b)). Therefore, we further confirmed the potential role of JNK in FAF1-meditated hepatic metabolic perturbation. We used the JNK inhibitor SP600125 to block JNK phosphorylation and found that SP600125 successfully reversed the effect of FAF1 overexpression on the insulin signaling pathway and the expression of IRS1 (Figures 5(c) and 5(d)). These data indicated that FAF1 mediated hepatic metabolic perturbation by way of the JNK signaling pathway.

### 3.6. FAF1 Physically Interacts with JNK and Actives the Activity of JNK

**Figure 6:**
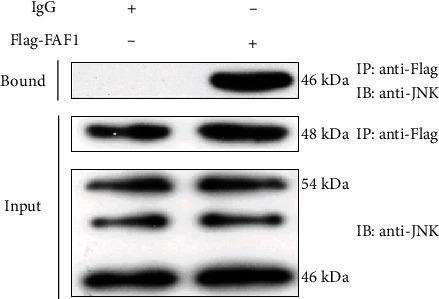
FAF1 physically interacted with JNK. Cells were transfected with Flag-tagged FAF1 plasmid. After immunoprecipitation with the anti-Flag antibody, coimmunoprecipitated proteins were detected by using the anti-JNK antibody.

Based on the result that FAF1 overexpression activated the JNK phosphorylation, we asked whether FAF1 interacted with JNK to regulate its activity. We next investigated the link between FAF1 and JNK. HepG2 cells transfected with Flag-tagged FAF1 were harvested, and cell lysates were immunoprecipitated with an anti-FAF1 antibody, followed by immunoblotting with antibodies against the JNK. The result indicated that FAF1 was coimmunoprecipitated with JNK in HepG2 cells (Figure 6). Collectively, these data indicated that FAF1 interacted with JNK to involve in the insulin signaling pathway.

## 4. Discussion

**Figure 7:**
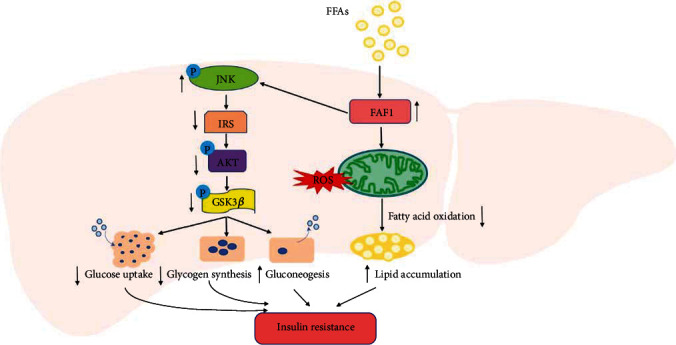
Proposed mechanisms of the role of FAF1 in hepatic insulin resistance.

Although it is well accepted that FAF1 has a vital role in various biological processes, its underlying mechanism in metabolic disorders and insulin resistance remains unclear. In the present study, we identified FAF1 as a novel regulator in hepatic insulin resistance. The key findings of our study are summarized in Figure 7. Our findings showed that the expression of FAF1 was increased in the livers of insulin-resistant rats, and hepatocytes treated with PA. Overexpression of FAF1 inhibited insulin-stimulated glucose uptake and glycogen synthesis, increased ROS production, and aggravated PA-induced lipid accumulation, whereas knockdown of FAF1 ameliorated these processes. Furthermore, we also found that FAF1 impaired the insulin signaling pathway via interacting with JNK to activate JNK phosphorylation and reduce the expression of IRS1.

Several studies revealed that FAF1 variations were susceptibility loci associated with type 2 diabetes [20, 21], but whether the expression of FAF1 was associated with the progression of type 2 diabetes was unclear. Kumar et al. found that circulating soluble apoptotic marker Fas was positively with insulin resistance in the serum of newly diagnosed type 2 diabetes [7]. Coincidentally, our results indicated the expression of FAF1 was positively with HOMA-IR and was clearly increased in insulin-resistant rats.

Compelling evidence identified that ROS, mainly produced by mitochondria, was closely associated with insulin resistance in humans and rodent models [30, 33, 34]. Our study found that FAF1 overexpression increased ROS production, which might be involved in insulin resistance. Noteworthily, FAF1 overexpression did not affect the expression of lipogenic transcription factor sterol regulatory element-binding protein 1 (SREBP1) but exacerbated PA-induced lipid accumulation, suggesting that FAF1 overexpression might impair mitochondrial function and lipid oxidation. In agreement with this, hepatic Fas overexpression in chow-fed mice impaired fatty acid oxidation and mitochondrial respiration, promoting hepatic lipid accumulation and insulin resistance [9]. Further studies to clarify the link between FAF1 and mitochondrial function are clearly warranted.

JNK, a member of the MAPK family, is usually activated by metabolic and inflammatory factors. Consistent with our findings, previous studies have indicated that hyperactivation of JNK plays a central role in metabolic disorder and insulin resistance [32, 35]. Sul et al. found that FAF1 activated JNK in the dopaminergic neurodegeneration [12]. However, the correlation of FAF1 with JNK in hepatic insulin resistance has not been investigated. Here, we reported that FAF1 physically interacted with JNK and was mainly responsible for JNK activation. Using a specific JNK inhibitor (SP600125) that could block the kinase activity of JNK, we demonstrated a beneficial effect of JNK inhibition on the insulin signaling pathway and an indispensable role of its activation in FAF1-exacerbated insulin resistance.

## 5. Conclusion

In conclusion, our study elucidates the biological effects and underlying mechanism of FAF1 in metabolic disorder and insulin resistance. FAF1 mediated hepatic metabolic disorder via interaction with JNK and impairment of the insulin signaling pathway, thereby leading to aggravated glucose metabolism disorder, lipid metabolism disorder, and insulin resistance.

## Data Availability

The data used to support the findings of this study are available from the corresponding author upon request.

## Supplementary Materials

Supplementary Materials Supplementary Table 1: primer sequences. Supplementary Table 2: sequence of small interfering RNA (siRNA) against FAF1 and negative control.
