# Supplementary material for: Fas-Associated Factor 1 Promotes Hepatic Insulin Resistance via JNK Signaling Pathway
Source: Oxid Med Cell Longev. 2021 Jan 16;2021:3756925. doi: 10.1155/2021/3756925 (PMC7826235; doi:10.1155/2021/3756925)
Supplement: Supplementary Materials — Supplementary Table 1: primer sequences. Supplementary Table 2: sequence of small interfering RNA (siRNA) against FAF1 and negative control. [file 3756925.f1.docx]

**Supplementary Table 1. Primer sequences**

| Genes | Sequences |
| --- | --- |
| h-*FAF1* | F: TGGAAGACAGTACGGTCCTAAA |
|  | R: GCACCAGCATGACTAGATGATG |
| h-*GAPDH* | F: CTGGGCTACACTGAGCACC |
|  | R: AAGTGGTCGTTGAGGGCAATG |
| h-*SREBP1* | F: ACAGTGACTTCCCTGGCCTAT |
|  | R: GCATGGACGGGTACATCTTCAA |
| h-*G6PC* | F: GTGTCCGTGATCGCAGACC |
|  | R: GACGAGGTTGAGCCAGTCTC |
| h-*PEPCK* | F: GGCTGAGAATACTGCCACACT |
|  | R: ACCGTCTTGCTCTCTACTCGT |
| h-*GLUT2* | F: GCCTGGTTCCTATGTATATCGGT |
|  | R: GCCACAGATCATAATTGCCCAAG |

**Supplementary Table 2. Sequence of small interfering RNA (siRNA) against FAF1 and negative control**

| siRNA | Sequence |
| --- | --- |
| FAF1 siRNA1 | F: GCAGCUAUCAAUGGUGUAATT |
|  | R: UUACACCAUUGAUAGCUGCTT |
| FAF1 siRNA2 | F: GGAACAGUCGGAAGAACAATT |
|  | R: UUGUUCUUCCGACUGUUCCTT |
| Negative control | F: UUCUCCGAACGUGUCACGUTT |
|  | R: ACGUGACACGUUCGGAGAATT |
